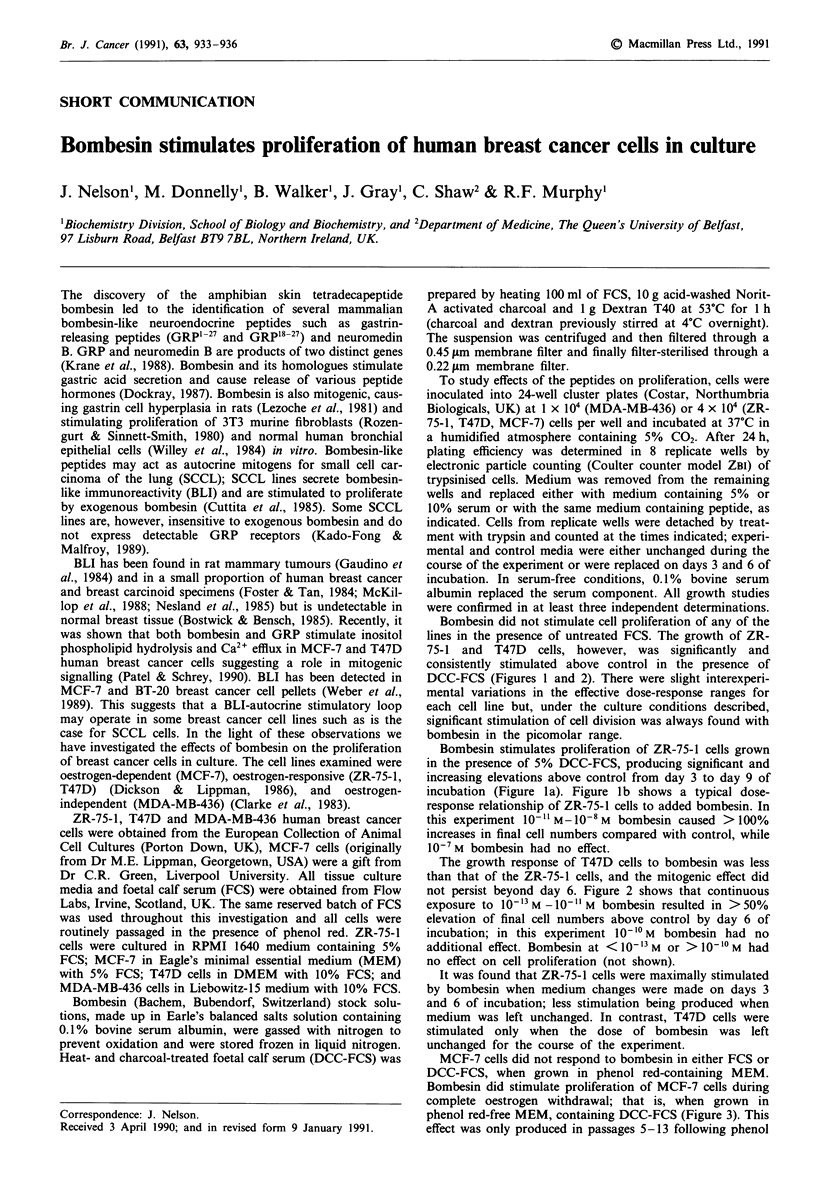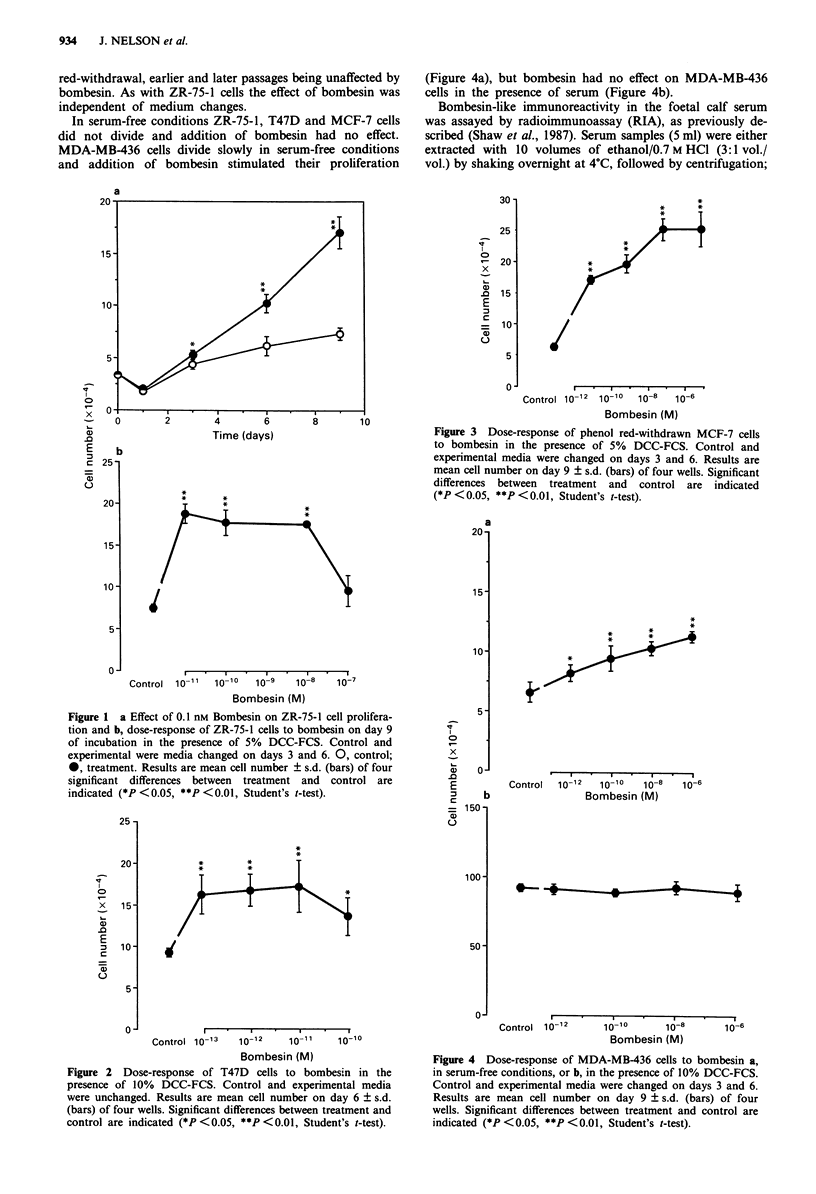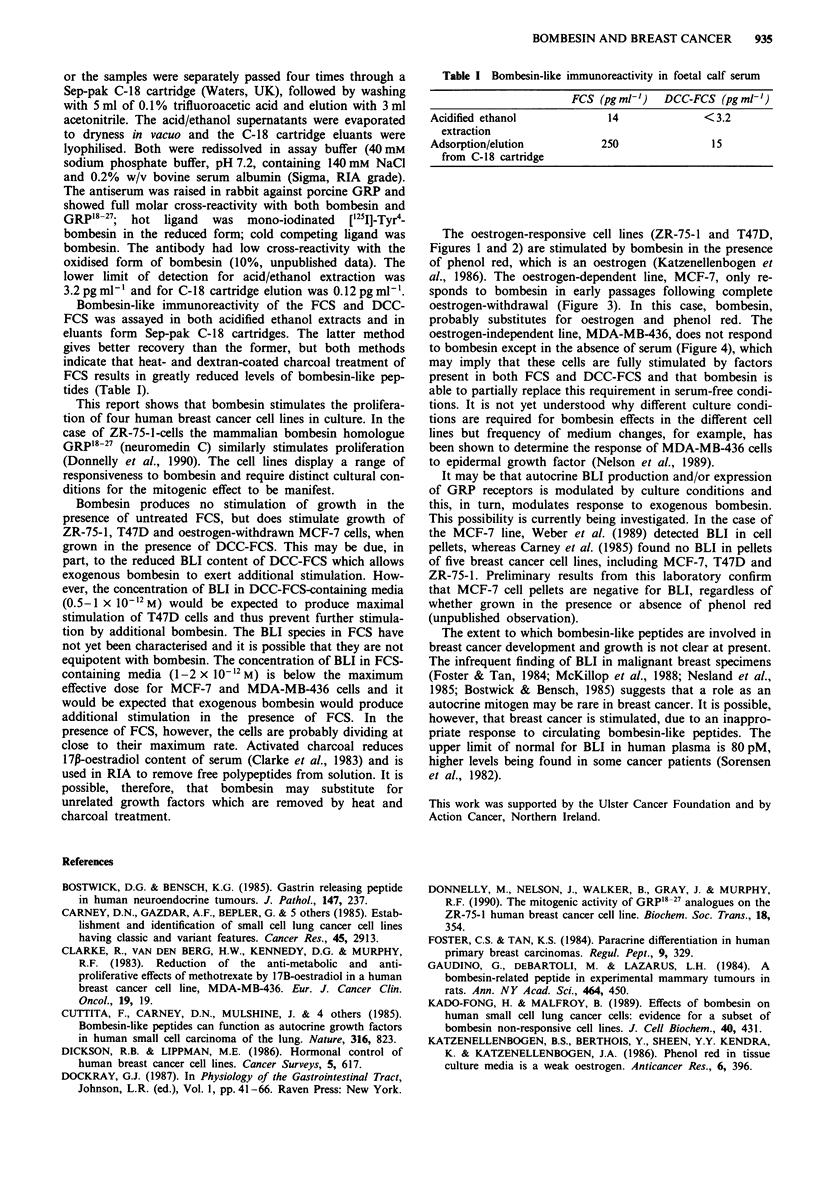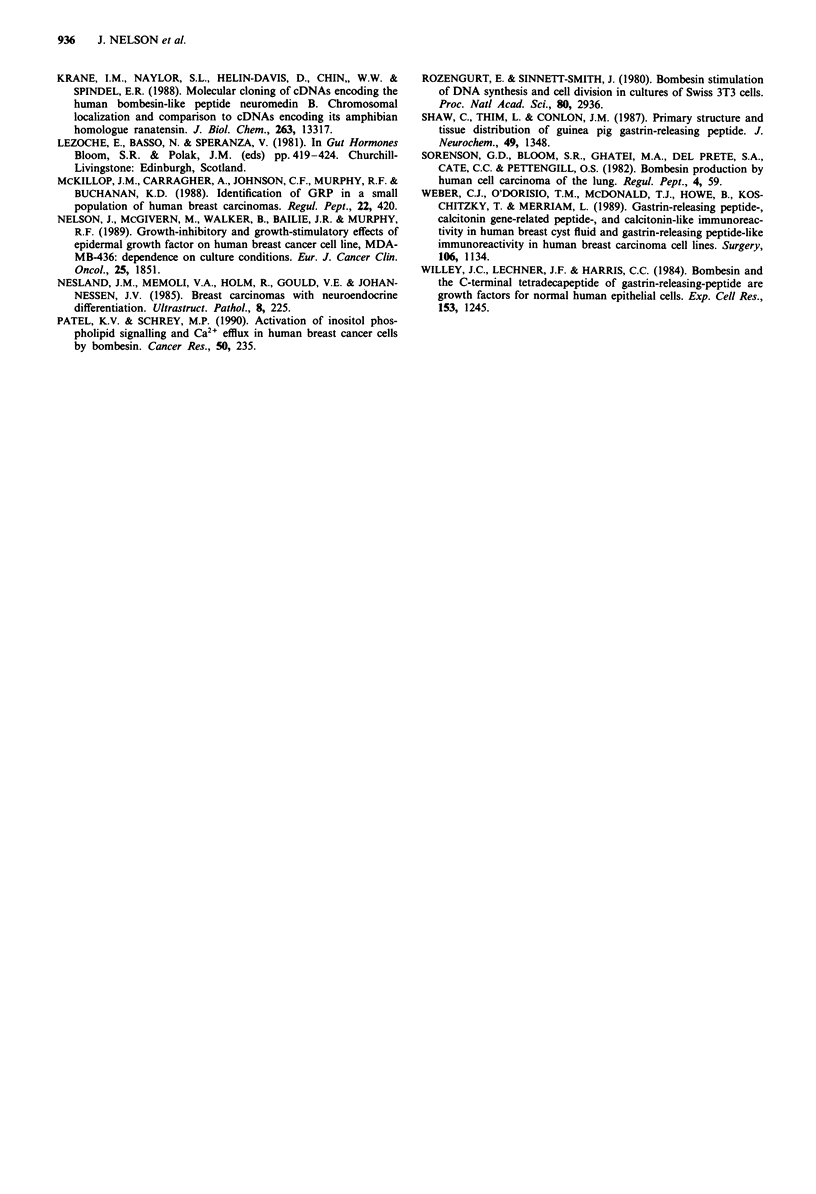# Bombesin stimulates proliferation of human breast cancer cells in culture.

**DOI:** 10.1038/bjc.1991.204

**Published:** 1991-06

**Authors:** J. Nelson, M. Donnelly, B. Walker, J. Gray, C. Shaw, R. F. Murphy

**Affiliations:** Biochemistry Division, School of Biology and Biochemistry, Queen's University of Belfast, Northern Ireland, UK.


Br. J. Cancer (1991), 63, 933-936 © Macmillan Press Ltd., 1991

SHORT COMMUNICATION

### Bombesin stimulates proliferation of human breast cancer cells in culture

J. Nelson[1], M. Donnelly[1], B. Walker[1], J. Gray[1], C. Shaw[2] & R.F. Murphy[1]

[1]*Biochemistry Division, School of Biology and Biochemistry, and* [2]*Department of Medicine, The Queen's University of Belfast, 97 Lisburn Road, Belfast BT9 7BL, Northern Ireland, UK.*

The discovery of the amphibian skin tetradecapeptide bombesin led to the identification of several mammalian bombesin-like neuroendocrine peptides such as gastrin-releasing peptides ($GRP^{1-27}$ and $GRP^{18-27}$) and neuromedin B. GRP and neuromedin B are products of two distinct genes (Krane *et al.*, 1988). Bombesin and its homologues stimulate gastric acid secretion and cause release of various peptide hormones (Dockray, 1987). Bombesin is also mitogenic, causing gastrin cell hyperplasia in rats (Lezoche *et al.*, 1981) and stimulating proliferation of 3T3 murine fibroblasts (Rozengurt & Sinnett-Smith, 1980) and normal human bronchial epithelial cells (Willey *et al.*, 1984) *in vitro*. Bombesin-like peptides may act as autocrine mitogens for small cell carcinoma of the lung (SCCL); SCCL lines secrete bombesin-like immunoreactivity (BLI) and are stimulated to proliferate by exogenous bombesin (Cuttita *et al.*, 1985). Some SCCL lines are, however, insensitive to exogenous bombesin and do not express detectable GRP receptors (Kado-Fong & Malfroy, 1989).

BLI has been found in rat mammary tumours (Gaudino *et al.*, 1984) and in a small proportion of human breast cancer and breast carcinoid specimens (Foster & Tan, 1984; McKillop *et al.*, 1988; Nesland *et al.*, 1985) but is undetectable in normal breast tissue (Bostwick & Bensch, 1985). Recently, it was shown that both bombesin and GRP stimulate inositol phospholipid hydrolysis and $Ca^{2+}$ efflux in MCF-7 and T47D human breast cancer cells suggesting a role in mitogenic signalling (Patel & Schrey, 1990). BLI has been detected in MCF-7 and BT-20 breast cancer cell pellets (Weber *et al.*, 1989). This suggests that a BLI-autocrine stimulatory loop may operate in some breast cancer cell lines such as is the case for SCCL cells. In the light of these observations we have investigated the effects of bombesin on the proliferation of breast cancer cells in culture. The cell lines examined were oestrogen-dependent (MCF-7), oestrogen-responsive (ZR-75-1, T47D) (Dickson & Lippman, 1986), and oestrogen-independent (MDA-MB-436) (Clarke *et al.*, 1983).

ZR-75-1, T47D and MDA-MB-436 human breast cancer cells were obtained from the European Collection of Animal Cell Cultures (Porton Down, UK), MCF-7 cells (originally from Dr M.E. Lippman, Georgetown, USA) were a gift from Dr C.R. Green, Liverpool University. All tissue culture media and foetal calf serum (FCS) were obtained from Flow Labs, Irvine, Scotland, UK. The same reserved batch of FCS was used throughout this investigation and all cells were routinely passaged in the presence of phenol red. ZR-75-1 cells were cultured in RPMI 1640 medium containing 5% FCS; MCF-7 in Eagle's minimal essential medium (MEM) with 5% FCS; T47D cells in DMEM with 10% FCS; and MDA-MB-436 cells in Liebowitz-15 medium with 10% FCS.

Bombesin (Bachem, Bubendorf, Switzerland) stock solutions, made up in Earle's balanced salts solution containing 0.1% bovine serum albumin, were gassed with nitrogen to prevent oxidation and were stored frozen in liquid nitrogen. Heat- and charcoal-treated foetal calf serum (DCC-FCS) was prepared by heating 100 ml of FCS, 10 g acid-washed Norit-A activated charcoal and 1 g Dextran T40 at 53°C for 1 h (charcoal and dextran previously stirred at 4°C overnight). The suspension was centrifuged and then filtered through a 0.45 $\mu$m membrane filter and finally filter-sterilised through a 0.22 $\mu$m membrane filter.

To study effects of the peptides on proliferation, cells were inoculated into 24-well cluster plates (Costar, Northumbria Biologicals, UK) at $1 \times 10^4$ (MDA-MB-436) or $4 \times 10^4$ (ZR-75-1, T47D, MCF-7) cells per well and incubated at 37°C in a humidified atmosphere containing 5% $CO_2$. After 24 h, plating efficiency was determined in 8 replicate wells by electronic particle counting (Coulter counter model ZBI) of trypsinised cells. Medium was removed from the remaining wells and replaced either with medium containing 5% or 10% serum or with the same medium containing peptide, as indicated. Cells from replicate wells were detached by treatment with trypsin and counted at the times indicated; experimental and control media were either unchanged during the course of the experiment or were replaced on days 3 and 6 of incubation. In serum-free conditions, 0.1% bovine serum albumin replaced the serum component. All growth studies were confirmed in at least three independent determinations.

Bombesin did not stimulate cell proliferation of any of the lines in the presence of untreated FCS. The growth of ZR-75-1 and T47D cells, however, was significantly and consistently stimulated above control in the presence of DCC-FCS (Figures 1 and 2). There were slight interexperimental variations in the effective dose-response ranges for each cell line but, under the culture conditions described, significant stimulation of cell division was always found with bombesin in the picomolar range.

Bombesin stimulates proliferation of ZR-75-1 cells grown in the presence of 5% DCC-FCS, producing significant and increasing elevations above control from day 3 to day 9 of incubation (Figure 1a). Figure 1b shows a typical dose-response relationship of ZR-75-1 cells to added bombesin. In this experiment $10^{-11}$ M$-10^{-8}$ M bombesin caused $>100\%$ increases in final cell numbers compared with control, while $10^{-7}$ M bombesin had no effect.

The growth response of T47D cells to bombesin was less than that of the ZR-75-1 cells, and the mitogenic effect did not persist beyond day 6. Figure 2 shows that continuous exposure to $10^{-13}$ M $-10^{-11}$ M bombesin resulted in $>50\%$ elevation of final cell numbers above control by day 6 of incubation; in this experiment $10^{-10}$ M bombesin had no additional effect. Bombesin at $<10^{-13}$ M or $>10^{-10}$ M had no effect on cell proliferation (not shown).

It was found that ZR-75-1 cells were maximally stimulated by bombesin when medium changes were made on days 3 and 6 of incubation; less stimulation being produced when medium was left unchanged. In contrast, T47D cells were stimulated only when the dose of bombesin was left unchanged for the course of the experiment.

MCF-7 cells did not respond to bombesin in either FCS or DCC-FCS, when grown in phenol red-containing MEM. Bombesin did stimulate proliferation of MCF-7 cells during complete oestrogen withdrawal; that is, when grown in phenol red-free MEM, containing DCC-FCS (Figure 3). This effect was only produced in passages 5–13 following phenol

Correspondence: J. Nelson.
Received 3 April 1990; and in revised form 9 January 1991.

red-withdrawal, earlier and later passages being unaffected by bombesin. As with ZR-75-1 cells the effect of bombesin was independent of medium changes.

In serum-free conditions ZR-75-1, T47D and MCF-7 cells did not divide and addition of bombesin had no effect. MDA-MB-436 cells divide slowly in serum-free conditions and addition of bombesin stimulated their proliferation (Figure 4a), but bombesin had no effect on MDA-MB-436 cells in the presence of serum (Figure 4b).

Bombesin-like immunoreactivity in the foetal calf serum was assayed by radioimmunoassay (RIA), as previously described (Shaw *et al.*, 1987). Serum samples (5 ml) were either extracted with 10 volumes of ethanol/0.7 M HCl (3:1 vol./vol.) by shaking overnight at 4°C, followed by centrifugation;

**Figure 1** **a** Effect of 0.1 nM Bombesin on ZR-75-1 cell proliferation and **b**, dose-response of ZR-75-1 cells to bombesin on day 9 of incubation in the presence of 5% DCC-FCS. Control and experimental were media changed on days 3 and 6. O, control; ●, treatment. Results are mean cell number ± s.d. (bars) of four significant differences between treatment and control are indicated (*$P < 0.05$, **$P < 0.01$, Student's *t*-test).

**Figure 2** Dose-response of T47D cells to bombesin in the presence of 10% DCC-FCS. Control and experimental media were unchanged. Results are mean cell number on day 6 ± s.d. (bars) of four wells. Significant differences between treatment and control are indicated (*$P < 0.05$, **$P < 0.01$, Student's *t*-test).

**Figure 3** Dose-response of phenol red-withdrawn MCF-7 cells to bombesin in the presence of 5% DCC-FCS. Control and experimental media were changed on days 3 and 6. Results are mean cell number on day 9 ± s.d. (bars) of four wells. Significant differences between treatment and control are indicated (*$P < 0.05$, **$P < 0.01$, Student's *t*-test).

**Figure 4** Dose-response of MDA-MB-436 cells to bombesin **a**, in serum-free conditions, or **b**, in the presence of 10% DCC-FCS. Control and experimental media were changed on days 3 and 6. Results are mean cell number on day 9 ± s.d. (bars) of four wells. Significant differences between treatment and control are indicated (*$P < 0.05$, **$P < 0.01$, Student's *t*-test).

or the samples were separately passed four times through a Sep-pak C-18 cartridge (Waters, UK), followed by washing with 5 ml of 0.1% trifluoroacetic acid and elution with 3 ml acetonitrile. The acid/ethanol supernatants were evaporated to dryness *in vacuo* and the C-18 cartridge eluants were lyophilised. Both were redissolved in assay buffer (40 mM sodium phosphate buffer, pH 7.2, containing 140 mM NaCl and 0.2% w/v bovine serum albumin (Sigma, RIA grade). The antiserum was raised in rabbit against porcine GRP and showed full molar cross-reactivity with both bombesin and $GRP^{18-27}$; hot ligand was mono-iodinated $[^{125}I]$-$Tyr^4$-bombesin in the reduced form; cold competing ligand was bombesin. The antibody had low cross-reactivity with the oxidised form of bombesin (10%, unpublished data). The lower limit of detection for acid/ethanol extraction was 3.2 pg $ml^{-1}$ and for C-18 cartridge elution was 0.12 pg $ml^{-1}$.

Bombesin-like immunoreactivity of the FCS and DCC-FCS was assayed in both acidified ethanol extracts and in eluants form Sep-pak C-18 cartridges. The latter method gives better recovery than the former, but both methods indicate that heat- and dextran-coated charcoal treatment of FCS results in greatly reduced levels of bombesin-like peptides (Table I).

This report shows that bombesin stimulates the proliferation of four human breast cancer cell lines in culture. In the case of ZR-75-1-cells the mammalian bombesin homologue $GRP^{18-27}$ (neuromedin C) similarly stimulates proliferation (Donnelly *et al.*, 1990). The cell lines display a range of responsiveness to bombesin and require distinct cultural conditions for the mitogenic effect to be manifest.

Bombesin produces no stimulation of growth in the presence of untreated FCS, but does stimulate growth of ZR-75-1, T47D and oestrogen-withdrawn MCF-7 cells, when grown in the presence of DCC-FCS. This may be due, in part, to the reduced BLI content of DCC-FCS which allows exogenous bombesin to exert additional stimulation. However, the concentration of BLI in DCC-FCS-containing media ($0.5-1 \times 10^{-12}$ M) would be expected to produce maximal stimulation of T47D cells and thus prevent further stimulation by additional bombesin. The BLI species in FCS have not yet been characterised and it is possible that they are not equipotent with bombesin. The concentration of BLI in FCS-containing media ($1-2 \times 10^{-12}$ M) is below the maximum effective dose for MCF-7 and MDA-MB-436 cells and it would be expected that exogenous bombesin would produce additional stimulation in the presence of FCS. In the presence of FCS, however, the cells are probably dividing at close to their maximum rate. Activated charcoal reduces 17β-oestradiol content of serum (Clarke *et al.*, 1983) and is used in RIA to remove free polypeptides from solution. It is possible, therefore, that bombesin may substitute for unrelated growth factors which are removed by heat and charcoal treatment.

**Table I** Bombesin-like immunoreactivity in foetal calf serum

| | *FCS* (pg $ml^{-1}$) | *DCC-FCS* (pg $ml^{-1}$) |
|---|---|---|
| Acidified ethanol extraction | 14 | <3.2 |
| Adsorption/elution from C-18 cartridge | 250 | 15 |

The oestrogen-responsive cell lines (ZR-75-1 and T47D, Figures 1 and 2) are stimulated by bombesin in the presence of phenol red, which is an oestrogen (Katzenellenbogen *et al.*, 1986). The oestrogen-dependent line, MCF-7, only responds to bombesin in early passages following complete oestrogen-withdrawal (Figure 3). In this case, bombesin, probably substitutes for oestrogen and phenol red. The oestrogen-independent line, MDA-MB-436, does not respond to bombesin except in the absence of serum (Figure 4), which may imply that these cells are fully stimulated by factors present in both FCS and DCC-FCS and that bombesin is able to partially replace this requirement in serum-free conditions. It is not yet understood why different culture conditions are required for bombesin effects in the different cell lines but frequency of medium changes, for example, has been shown to determine the response of MDA-MB-436 cells to epidermal growth factor (Nelson *et al.*, 1989).

It may be that autocrine BLI production and/or expression of GRP receptors is modulated by culture conditions and this, in turn, modulates response to exogenous bombesin. This possibility is currently being investigated. In the case of the MCF-7 line, Weber *et al.* (1989) detected BLI in cell pellets, whereas Carney *et al.* (1985) found no BLI in pellets of five breast cancer cell lines, including MCF-7, T47D and ZR-75-1. Preliminary results from this laboratory confirm that MCF-7 cell pellets are negative for BLI, regardless of whether grown in the presence or absence of phenol red (unpublished observation).

The extent to which bombesin-like peptides are involved in breast cancer development and growth is not clear at present. The infrequent finding of BLI in malignant breast specimens (Foster & Tan, 1984; McKillop *et al.*, 1988; Nesland *et al.*, 1985; Bostwick & Bensch, 1985) suggests that a role as an autocrine mitogen may be rare in breast cancer. It is possible, however, that breast cancer is stimulated, due to an inappropriate response to circulating bombesin-like peptides. The upper limit of normal for BLI in human plasma is 80 pM, higher levels being found in some cancer patients (Sorensen *et al.*, 1982).

This work was supported by the Ulster Cancer Foundation and by Action Cancer, Northern Ireland.

### References

BOSTWICK, D.G. & BENSCH, K.G. (1985). Gastrin releasing peptide in human neuroendocrine tumours. *J. Pathol.*, **147**, 237.

CARNEY, D.N., GAZDAR, A.F., BEPLER, G. & 5 others (1985). Establishment and identification of small cell lung cancer cell lines having classic and variant features. *Cancer Res.*, **45**, 2913.

CLARKE, R., VAN DEN BERG, H.W., KENNEDY, D.G. & MURPHY, R.F. (1983). Reduction of the anti-metabolic and anti-proliferative effects of methotrexate by 17B-oestradiol in a human breast cancer cell line, MDA-MB-436. *Eur. J. Cancer Clin. Oncol.*, **19**, 19.

CUTTITA, F., CARNEY, D.N., MULSHINE, J. & 4 others (1985). Bombesin-like peptides can function as autocrine growth factors in human small cell carcinoma of the lung. *Nature*, **316**, 823.

DICKSON, R.B. & LIPPMAN, M.E. (1986). Hormonal control of human breast cancer cell lines. *Cancer Surveys*, **5**, 617.

DOCKRAY, G.J. (1987). In *Physiology of the Gastrointestinal Tract*, Johnson, L.R. (ed.), Vol. 1, pp. 41-66. Raven Press: New York.

DONNELLY, M., NELSON, J., WALKER, B., GRAY, J. & MURPHY, R.F. (1990). The mitogenic activity of $GRP^{18-27}$ analogues on the ZR-75-1 human breast cancer cell line. *Biochem. Soc. Trans.*, **18**, 354.

FOSTER, C.S. & TAN, K.S. (1984). Paracrine differentiation in human primary breast carcinomas. *Regul. Pept.*, **9**, 329.

GAUDINO, G., DEBARTOLI, M. & LAZARUS, L.H. (1984). A bombesin-related peptide in experimental mammary tumours in rats. *Ann. NY Acad. Sci.*, **464**, 450.

KADO-FONG, H. & MALFROY, B. (1989). Effects of bombesin on human small cell lung cancer cells: evidence for a subset of bombesin non-responsive cell lines. *J. Cell Biochem.*, **40**, 431.

KATZENELLENBOGEN, B.S., BERTHOIS, Y., SHEEN, Y.Y. KENDRA, K. & KATZENELLENBOGEN, J.A. (1986). Phenol red in tissue culture media is a weak oestrogen. *Anticancer Res.*, **6**, 396.

KRANE, I.M., NAYLOR, S.L., HELIN-DAVIS, D., CHIN,, W.W. & SPINDEL, E.R. (1988). Molecular cloning of cDNAs encoding the human bombesin-like peptide neuromedin B. Chromosomal localization and comparison to cDNAs encoding its amphibian homologue ranatensin. *J. Biol. Chem.*, **263**, 13317.

LEZOCHE, E., BASSO, N. & SPERANZA, V. (1981). In *Gut Hormones* Bloom, S.R. & Polak, J.M. (eds) pp. 419–424. Churchill-Livingstone: Edinburgh, Scotland.

MCKILLOP, J.M., CARRAGHER, A., JOHNSON, C.F., MURPHY, R.F. & BUCHANAN, K.D. (1988). Identification of GRP in a small population of human breast carcinomas. *Regul. Pept.*, **22**, 420.

NELSON, J., MCGIVERN, M., WALKER, B., BAILIE, J.R. & MURPHY, R.F. (1989). Growth-inhibitory and growth-stimulatory effects of epidermal growth factor on human breast cancer cell line, MDA-MB-436: dependence on culture conditions. *Eur. J. Cancer Clin. Oncol.*, **25**, 1851.

NESLAND, J.M., MEMOLI, V.A., HOLM, R., GOULD, V.E. & JOHANNESSEN, J.V. (1985). Breast carcinomas with neuroendocrine differentiation. *Ultrastruct. Pathol.*, **8**, 225.

PATEL, K.V. & SCHREY, M.P. (1990). Activation of inositol phospholipid signalling and $Ca^{2+}$ efflux in human breast cancer cells by bombesin. *Cancer Res.*, **50**, 235.

ROZENGURT, E. & SINNETT-SMITH, J. (1980). Bombesin stimulation of DNA synthesis and cell division in cultures of Swiss 3T3 cells. *Proc. Natl Acad. Sci.*, **80**, 2936.

SHAW, C., THIM, L. & CONLON, J.M. (1987). Primary structure and tissue distribution of guinea pig gastrin-releasing peptide. *J. Neurochem.*, **49**, 1348.

SORENSON, G.D., BLOOM, S.R., GHATEI, M.A., DEL PRETE, S.A., CATE, C.C. & PETTENGILL, O.S. (1982). Bombesin production by human cell carcinoma of the lung. *Regul. Pept.*, **4**, 59.

WEBER, C.J., O'DORISIO, T.M., MCDONALD, T.J., HOWE, B., KOSCHITZKY, T. & MERRIAM, L. (1989). Gastrin-releasing peptide-, calcitonin gene-related peptide-, and calcitonin-like immunoreactivity in human breast cyst fluid and gastrin-releasing peptide-like immunoreactivity in human breast carcinoma cell lines. *Surgery*, **106**, 1134.

WILLEY, J.C., LECHNER, J.F. & HARRIS, C.C. (1984). Bombesin and the C-terminal tetradecapeptide of gastrin-releasing-peptide are growth factors for normal human epithelial cells. *Exp. Cell Res.*, **153**, 1245.